# APOBEC3B: Future direction of liver cancer research

**DOI:** 10.3389/fonc.2022.996115

**Published:** 2022-09-20

**Authors:** Xingyue Yang, Jing Dai, Shun Yao, Jiaxing An, Guorong Wen, Hai Jin, Li Zhang, Liming Zheng, Xingyue Chen, Zhiqiang Yi, Biguang Tuo

**Affiliations:** ^1^ Department of Gastroenterology, Digestive Disease Hospital, Affiliated Hospital of Zunyi Medical University, Zunyi, China; ^2^ The Collaborative Innovation Center of Tissue Damage Repair and Regenerative Medicine of Zunyi Medical University, Zunyi, China

**Keywords:** liver cancer, hbv, APOBEC3B, APOBEC family, NF-κB signaling pathways

## Abstract

Liver cancer is one of the most common cancers in the world, and the rate of liver cancer is high due to the of its illness. The main risk factor for liver cancer is infection with the hepatitis B virus (HBV), but a considerable number of genetic and epigenetic factors are also directly or indirectly involved in the underlying pathogenesis of liver cancer. In particular, the apolipoprotein B mRNA editing enzyme, catalytic peptide-like protein (APOBEC) family (DNA or mRNA editor family), which has been the focus of virology research for more than a decade, has been found to play a significant role in the occurrence and development of various cancers, providing a new direction for the research of liver cancer. APOBEC3B is a cytosine deaminase that controls a variety of biological processes, such as protein expression, innate immunity, and embryonic development, by participating in the process of cytidine deamination to uridine in DNA and RNA. In humans, APOBEC3B has long been known as a DNA editor for limiting viral replication and transcription. APOBEC3B is widely expressed at low levels in a variety of normal tissues and organs, but it is significantly upregulated in different types of tumor tissues and tumor lines. Thus, APOBEC3B has received increasing attention in various cancers, but the role of APOBEC3B in the occurrence and development of liver cancer due to infection with HBV remains unclear. This review provides a brief introduction to the pathogenesis of hepatocellular carcinoma induced by HBV, and it further explores the latest results of APOBEC3B research in the development of HBV and liver cancer, thereby providing new directions and strategies for the treatment and prevention of liver cancer.

## Introduction

Liver cancer is one of the most common cancers, and its fatality rate ranks fourth in the world (1) due to its high degree of malignancy and hidden disease symptoms. Hepatitis B virus (HBV) is the leading cause of liver cancer incidence and mortality globally, particularly in Africa and East Asia where 60% of cases are caused by HBV (2). HBV is a partially double-stranded DNA virus whose clinical outcomes of infection are influenced by viral replication and changes in the host immune system (2). The innate immune system is the first line of defense of the host immune system, and in recent years, a number of “limiting factors” with antiviral properties have also been discovered, including members of the apolipoprotein B mRNA editing enzyme, catalytic peptide-like protein (APOBEC) protein family (3, 4). The APOBEC family consists of 11 cytidine deaminase members in humans that function primarily by inducing DNA or RNA upper cytosine deamination mutations to defend against retroviral or retrotransposon transmission, and these members have been identified as widely involved in various antiviral processes. APOBEC3B is the most common member of the family, and it is involved in the development of cancer, including liver cancer (5), breast cancer (6), gastric cancer (7), chondrosarcoma (8), kidney cancer (9), colorectal cancer (10), cervical squamous cell carcinoma (11), lung cancer (12), and bladder cancer (13). In humans, APOBEC3B has also been reported to be significantly upregulated in liver cancer, but its role in liver cancer is unclear. In this article, we mainly review the latest research on the molecular mechanisms of APOBEC3B in limiting the pathogenesis of HBV infection and liver cancer to provide a new therapeutic direction or target for liver cancer treatment.

## Manuscript

### APOBEC3B: family, localization, structure, and function

**Figure 1:**
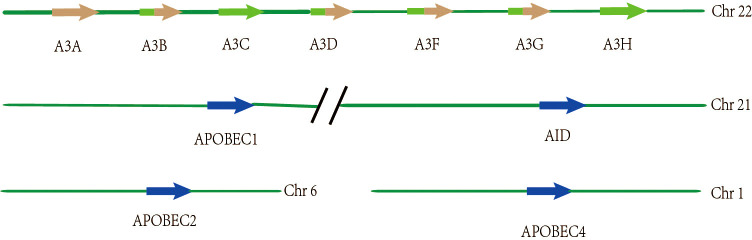
Spatial location distribution of genes among members of the APOBEC3 family. The seven members of APOBEC3 are arranged in tandem on chromosome 22, of which APOBEC3B, APOBEC3D, APOBEC3F, and APOBEC3G have two zinc catalytic enzyme structures, while the others have only one. APOBEC1 and AID are connected in tandem on chromosome 21, respectively. APOBEC2 and APOBEC4 are present on chromosome 6 and chromosome 1, respectively.

The APOBEC genes belong to a family of cytidine deaminases that deaminize cytidine to uridine on DNA or mRNA, and their high mutagenic activity limits viral reverse transcriptase and promotes instability in the genome of cancer cells (14). In humans, the APOBEC superfamily consists of 11 members, namely activation-induced cytosine deaminase (AID), APOBEC1 (A1), APOBEC2 (A2), APOBEC3A-H (A3A, A3B, A3C, A3D, A3F, A3G, and A3H), and APOBEC4 (A4), all of which play a key role in the biological processes of cells (**Table 1**). The chromosomal locations of the APOBEC family members are as follows: APOBEC3A-H are concatenated on chromosome 22; AID and APOBEC1 are concatenated on chromosome 12; APOBEC2 is localized on chromosome 6; and APOBEC4 is localized on chromosome 1, respectively (16, 17) (**Figure 1**). The genes in this family encode a cytidine deaminase, containing one or more conserved cytidine deaminase domains (CDAs), which are zinc-dependent catalytic domains that contain the common amino acid sequence of H-X-E-X23-28-P-CX2-4-C (X represents any amino acid) (18). These enzymes transmute DNA by a zinc-mediated hydrolysis mechanism to deaminize cytosine to uracil or convert cytosine to guanine (C to G) (19, 20). This pattern of mutations is the most common in cancer, second only to those that are attributed to aging (C-to-T in the CG dinucleotide motif, most likely due to water-mediated methylcytosine deamination). A1 was firstly discovered as an RNA-editing enzyme to deaminate at specific locations in mRNA to produce early stop codons, and it has been shown that A1 has strong deamination activity at the DNA level (21, 22). The function of A2 and A4 in humans remains unclear as no enzymatic activity has been demonstrated thus far. The seven A3 members are generally considered to be important barriers to viral replication and transcription, playing an important role in acquired immunity, unlike the basic function of AIDS, which is innate immunity (23). The A3 members are closely involved in immunity in a variety of ways and are a powerful force against endogenous and exogenous viruses. For example,A3G acts as a DNA editor to resist virus replication and transcription, and A3G also facilitates CD8+ cytotoxic T lymphocyte (CTL) recognition of infected T lymphocytes and limits marginal band B cells to transform rapid immune responses into more long-lasting B cell responses in germination centers (24). In addition, a recent study has suggested that inflammation-related factors induce A3A to edit the mRNAs of viral disease-causing genes in a large number of macrophages and monocytes, suggesting that A3A also plays an important role in the immune microenvironment (25). In particular, as the most involved member of this family in the cancer pathophysiology process, APOBEC3B limits viral reverse transcriptase by editing complementary DNA (cDNA) intermediates by relying on the action of cytidine deaminase (26), and it is also involved in promoting the biological occurrence and evolution of cancer through its role as a dependent or independent cytidine deaminase in the immune microenvironment (6, 11, 27).

**Table 1:** Genomic structure, function and sequence specificity of APOBE3C family (1, 15) (Partial data source: genecard).

Gene	Genetic information(location/exons)	Tissue expression	Cellular localization	Deaminase substrate	Target sequence(C=edit site)	Disorder
AID	12p13/5	Activated B cells	Cell wide	ssDNA, RNA	5’-WRC-3’ (W = A or T; R = A o r G)	None
APOBEC1	12p13.1/5	Gastrointestinal tract	Cell wide	ssDNA, RNA	5’-AC (n4–6) UGAUnnGnnnn-3’ (for n, A and U preferred)	Neurofibromatosis, Immunodeficiency with hyper-lgm
APOBEC2	6p21/3	Heart, skeletal muscle, TNF/activated liver cells	Cell wide	?	?	Immunodeficiency with hyper-lgm
	22q13.1/5	Monocytes/macrophages, non-progenitor cells	Cell wide	ssDNA, RNA	5’-TC-3’	Plantar wart, Bone leiomyosarcoma, Psoriasis
APOBEC3B	22q13.1/8	PKC-induced cancer cells, IFN/-activated liver cells	Nuclear	ssDNA	5’-TC-3’	Bone leiomyosarcoma, Recessive dystrophic epidermolysis bullosa
APOBEC3C	22q13.1/4	Immune centers, peripheral blood cells	Cell wide	ssDNA	5’-TC-3’	Plantar warts
APOBEC3D	22q13.1/7	Immune centers, peripheral blood cells	Cytoplasmic	ssDNA	5’-TC-3’	None
APOBEC3F	22q13.1/8	Immune centers, peripheral blood cells, IFN/-activated liver cells	Cytoplasmic	ssDNA	5’-TC-3’	Immune deficiency disease, Aids
APOBEC3G	22q13.1/8	Immune centers, peripheral blood cells, IFN/-activated liver cells	Cytoplasmic	ssDNA	5’-CC-3’,5’-TC-3’	Hepatitis B, Immunodeficiency disease, Aids
APOBEC3H	22q13.1/5	Immune centers, peripheral blood cells	Cell wide	ssDNA	5’-TC-3’	Plantar warts, Recessive dystrophic epidermolysis bullosa, Aids
APOBEC4	1q25.3/2	.	.	.	.	Immunodeficiency with hyper-lgm

Analysis of The Cancer Genome Atlas (TCGA) mutation data has shown that APOBEC cytidine deaminase mutation patterns lead to genomic instability and are thus involved in carcinogenic somatic mutations (28, 29). In this family, all APOBEC3s, except for APOBEC3B, APOBEC3D, APOBEC3F, and APOBEC3G, contain only one CDA, which may explain why APOBEC3B is an epigenetic factor in many cancers (**Figure 1**) (20, 30). It remains controversial whether the two CDA structures in APOBEC3B are deaminated. Recent studies have suggested that only the end of the carboxyl group in the CDA show deamination activity, while the CDA at the N-terminus does not have deamination activity (31). There is increasing evidence that APOBEC3B-catalyzed genomic cytidine deaminase mutation patterns are the leading cause of dispersion and aggregation mutations in variouscancers (11). In addition, the expression level of APOBEC3B in normal human organ tissues is relatively low, but the expression level of APOBEC3B in different tumors tissue and cell lines is abnormally elevated (32, 33). Moreover, the unique nuclear localization of APOBEC3B also facilitates mutations to promote tumor development (34). Previous studies have indicated that APOBEC3B is uniquely enriched in cervical, breast, lung, and bladder cancers (35). In particular, APOBEC3B has been shown to be a prognostic marker in estrogen receptor (ER)^+^ breast cancer, and that it plays a key role in the progression of breast cancer (7). Furthermore, APOBEC3B has been reported to be significantly elevated in liver cancer tissues relative to normal tissues, suggesting that it also plays an essential role in the occurrence and development of liver cancer (5). However, the role of APOBEC3B in liver cancer remains unclear, thereby requiring further investigation.

### HBV and liver cancer

Liver cancer is one of the most prevalent malignant tumors, and it has characteristics of late detection and high fatality rate (36). The progression of liver cancer is driven by a combination of factors including genetic predisposition, lifestyle and environmental factors, as well as chronic HBV and hepatitis C virus (HCV) infections. In particular, HBV infection is the most important cause of the high incidence of liver cancer in Asian populations (37, 38). There are approximately 240 million long-term HBV infections in the world, and uncontrolled chronic HBV infection can further evolve into life-threatening end-stage chronic liver diseases such as cirrhosis and liver cancer (39, 40). Previous studies have suggested that normal hepatocytes are be converted into hepatocellular carcinoma cells through chronic inflammation, DNA damage, chromosomal instability, epigenetic modification, and early neovascularization after HBV (41).

HBV, which belongs to the Hepadnaviridae family, is a relaxed circular double chain DNA virus with a molecular weight of 3.2Kb, and it replicates mainly through RNA intermediates (42, 43). In the human liver, 90% of the liver is comprised of hepatocytes, and HBV enters liver cells through Na^+^/taurocholate cotransporter polypeptide (NTCP) (44). With entry into hepatocytes, the relaxed circular DNA (RC DNA) of HBV is converted into covalent closed circular DNA (cccDNA) within the nucleus, which in turn evolves into a template for transcription of all viral RNAs (pregenomic RNA, pgRNA; and subgenomic RNA) (41). HBV synthesizes proteins, including P, core, precore, and X proteins (45). It is worth mentioning that P protein, also known as DNA polymerase, is translated from pgRNA and can function as reverse transcriptase, DNA-dependent DNA polymerase, and RNaseH (46). PgRNA is important in viral replication, and it can be reversed by the P protein into new viral rcDNA, which is then retransmitted into the nucleus to form a pool of cccDNA or secreted as an infectious virion with envelope proteins (47). In contrast, HBX (the X protein), as a key oncovirus gene protein, promotes the occurrence and development of hepatocellular carcinoma by regulating the cell cycle, cell signaling pathways, and DNA repair (48, 49). Surprisingly, HBX also participates in the replication of HBV by recruiting CBP, P300, and PCAF proteins into the hidden pathway of cccDNA (50).

HBV mainly drives the occurrence and development of liver cancer through indirect inflammatory liver damage and direct carcinogenic potential. Because HBV is a non-cytopathic virus, the specific immunity mediated by CD4^+^ and CD8^+^ T cells as well as the non-specific immunity produced by B cells are used to control the virus after invading the body, of which CD8^+^ T cells play a leading role (51–53). In addition, some cytokine responses (upregulation of IL-10 or TGF-β) regulatory T cells (Tregs) also play a role in liver damage (52, 54, 55). In acute injury, the liver can restore its initial liver function and size through regeneration of normal hepatocytes; however, chronic inflammatory liver injury and repeated compensatory proliferation of liver cells associated with persistent viral infection in chronic hepatitis B can lead to hepatic fibrosis or hepatic sclerosis and hepatocellular carcinoma (56, 57). Previous experiments have provided evidence for compensatory proliferation (58), chronic inflammation (52), and concomitant changes in molecular signaling pathways (59) associated with HBVinfection that drive the occurrence and development of liver cancer. However, occult HBV (the virus persists in the state of rcDNA or cccDNA in HBs Ag-negative patients) with the absence of inflammation, severe liver damage, and direct carcinogenic viral factors, or cancer supports the hypothesis of the direct carcinogenic potential of the HBV (60). The existing mainstream hypothesis about the direct carcinogenic mechanism of HBV mainly includes the following three aspects: a) HBV integrates tumor genes of the host through its own reverse transcription pathway; b) genomic is instability caused by HBV retroreplication when integrating the host genome group; and c) HBV directly affects cellular function or activates oncogenic signaling pathways through its own oncovirus gene proteins (HBx and PreS) (37). Hence, controlling HBV infection is important in the prevention and treatment of liver cancer.

### A molecular mechanism of APOBEC3B restriction in HBV virus infection

HBV is a partial double-stranded DNA virus that is replicated by retrotransmission within the cytoplasmic viral core particles, and its continuous replication within the host is a high-risk factor for the development of liver cancer. In the past, the treatment of HBV infection has involved the inhibition of its replication, resulting in reduced risk of liver sclerosis or liver cancer, but, cccDNA remains in the nucleus. Therefore, these treatments do not completely eliminate the infection of the HBV. Recent reports have suggested that the APOBEC3B cytidine deaminase family member is the “limiting factor” of HBV because it edits HBV cccDNA within the nuclear DNA, resulting in its degradation, which completely eliminates the HBV (61). Moreover, reports have suggested that lymphotoxin-β receptors upregulate APOBEC3B, resulting in cytidine deamination-dependent cccDNA degradation (62). Hence, it is important to explore the molecular mechanism of APOBEC3B in the degradation of ccc DNA to provide a new therapeutic direction for the treatment of hepatitis B.

**Figure 2:**
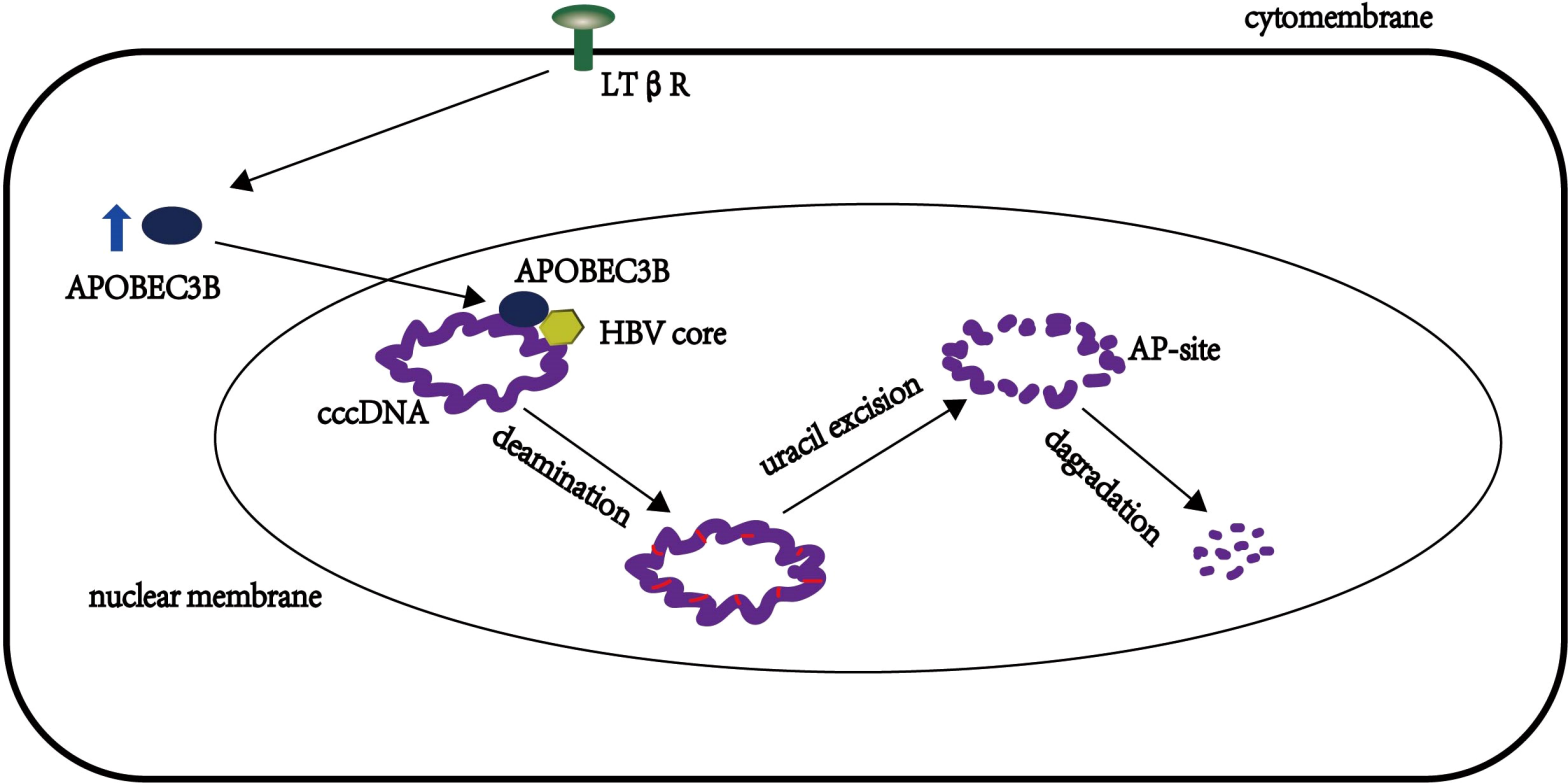
APOBEC3B-induced molecular mechanism model of degradation of HBV cccDNA.

APOBEC3 is involved primarily in antiviral molecular mechanisms through both dependent and independent deaminases (4). APOBEC3B has been reported to integrate HIV-1 particles in HIV viruses as a nucleoplasmic shuttle protein to limit its replication (63), but its mechanism remains unclear. Lucifora et al. proposed a mechanistic model of APOBEC3B degradation of ccc DNA, in which APOBEC3B participates in the degradation of cccDNA by co-localizing or interacting with HBV cores in the nucleus (62). ccc DNA is deaminated by APOBEC3B in a state of temporary single-stranded cc DNA, and DNA glycosyl deaminase then recognizes the uracils that excise ccc DNA to produce AP sites, which are then are recognized and degraded by AP endonucleases (64) (**Figure 2**). However, the APOBEC3B protein contains two cytidine deaminase structures, namely, CD1 and CD2, at the C terminus, and the antiviral activity of these two structures has been explored. Fu et al. demonstrated that H66 mutations in CD1 have no effect on deamination and antiviral activity, and they also demonstrated that H253 and D316 in the CD2 region are primarily responsible for this process (31, 65). Furthermore, Yanmeng et al. explored whether the APOBEC3B protein encodes HBV-related DNAs during reverse transcription in HBVs (65); they found that the antiviral mechanism of APOBEC3B differs from the activation-induced cytidine deaminase (AID), which primarily edits HBV RNA and single-stranded DNA during reverse transcription, while APOBEC3B primarily edits negative and positive stranded DNA of HBV (excluding pgRNA) (66). This protein can be in the nucleus or in the cytoplasm by interacting with the core protein or capsidprotein during the deamination process of DNA in the capsid, and DNA after deamination has more exposed AP sites, which are then recognized by DNA glycosylases and degraded by the base excision repair (BER) pathway (67). Thus, these findings suggest a new antiviral mechanism of APOBEC3B, involving core correlation in addition to targeting the degradation of ccc DNA within nuclear DNA.

There are still some unknown aspects of APOBEC3B in the mechanism of limiting HBV replication. For example, it is unknown why ccc DNA or associated DNA is degraded rather than repaired by cellular DNA repair mechanisms. Studies have reported that APOBEC3B also mutates the HBX protein of HBV to promote the development of liver cancer. Thus, it remains unclear whether, the mutation effect of APOBEC3B on HBV promotes liver cancer or inhibits liver cancer. Previous studies have suggested that cellular DNA repair mechanisms, such as UNG (uracil DNA glycosylase), resist the degradation mechanisms of ccc DNA by some APOBEC3B proteins but that these repair mechanisms do not affect the overall process of APOBEC3B restriction of HBV replication (68). In contrast, the basis for APOBEC3B mutation of the HBX protein of HBV is to promote the development of liver cancer in liver cancer cells (i.e., after liver cancer has occurred) (69), which does not contradict our hypothesis that APONEC3B inhibits the occurrence of liver cancer at the stage of HBV infection by degrading ccc DNA and DNA.

### APOBEC3B role in liver cancer and its molecular mechanisms

APOBEC3B, as a cytidine deaminase, has significant contributions to variouscancers (70). However, relatively little has been reported in highly fatal liver cancers, but there are still reports of APOBEC3B upregulation in liver cancer patients, contradicting the restriction of HBV infection and liver cancer by APOBEC3B *via* degrading the HBV genome (69, 71). Therefore, it is important to fully understand the role and molecular mechanisms of APOBEC3B in liver cancer. Scientists have also speculated whether APOBEC3B promotes the development of liver cancer by mutating the genome of liver cells, but previous studies have reported lack of mutation patterns of APOBEC3B in liver cancer (72). Although recent reported mutational signals for APOBEC3B during replication and transcription, overexpression of APOBEC3B does not affect genomic DNA (62, 73, 74). Therefore, further exploration of the role of APOBEC3B in liver cancer is required.

It is well known that the inflammatory process in the tumor microenvironment and the complex interaction between immune cells and cancer cells are considered to be the drivers and determinants of tumor disease outcome. In addition, the most common cause of hepatocellular carcinoma is underlying chronic liver inflammation and altered immune response (75). Recent studies have found that APOBEC3B provides a potential link among inflammation, cancer, and immune processes, which may be a novel finding for the non-enzyme-dependent function of APOBEC3B (76). There are many myeloid-derived suppressor cells (MDSCs) and tumor-associated macrophages (TAMs) in the microenvironment of liver cancer. These cells release oxidative molecules and stimulate other immunosuppressive cells to inhibit the function of CD8^+^ T cells, resulting in a better prognosis of liver cancer (77). Importantly, it has been reported that there is a positive feedback loop in the presence of chemotaxis between IL-6 and APOBEC3B in hepatoma cells (78), i.e., APOBEC3B promotes IL-6 to recruit MDSCs and TAMs to promote the development of liver cancer. Regarding molecular mechanisms of interaction between chronic hepatitis and liver cancer, it has been reported that triggering the classical and non-classical NF-κB signaling pathways leads to the promotion of chronic hepatitis to liver cancer (79, 80), such as the lymphatic toxin, LTα1β2, which is significantly upregulated in liver cancer. Moreover, Duowei et al. discovered and identified that nonclassical NF-κB signaling pathways stimulate the APOBEC3B-binding promoter through the RelB/p52 complex to increase the transcriptional expression of APOBEC3B, while an increase in the expression of APOBEC3B significantly increases the CCL2 chemokine, thus recruiting MDSCs and TAMs to contribute to the development of liver cancer (81). The role of APOBEC3B in inflammation, cancer, and immunity suggests that– APOBEC3B may be used as an immunomodulatory factor to promote tumor progression by altering the immune microenvironment (76).

**Figure 3:**
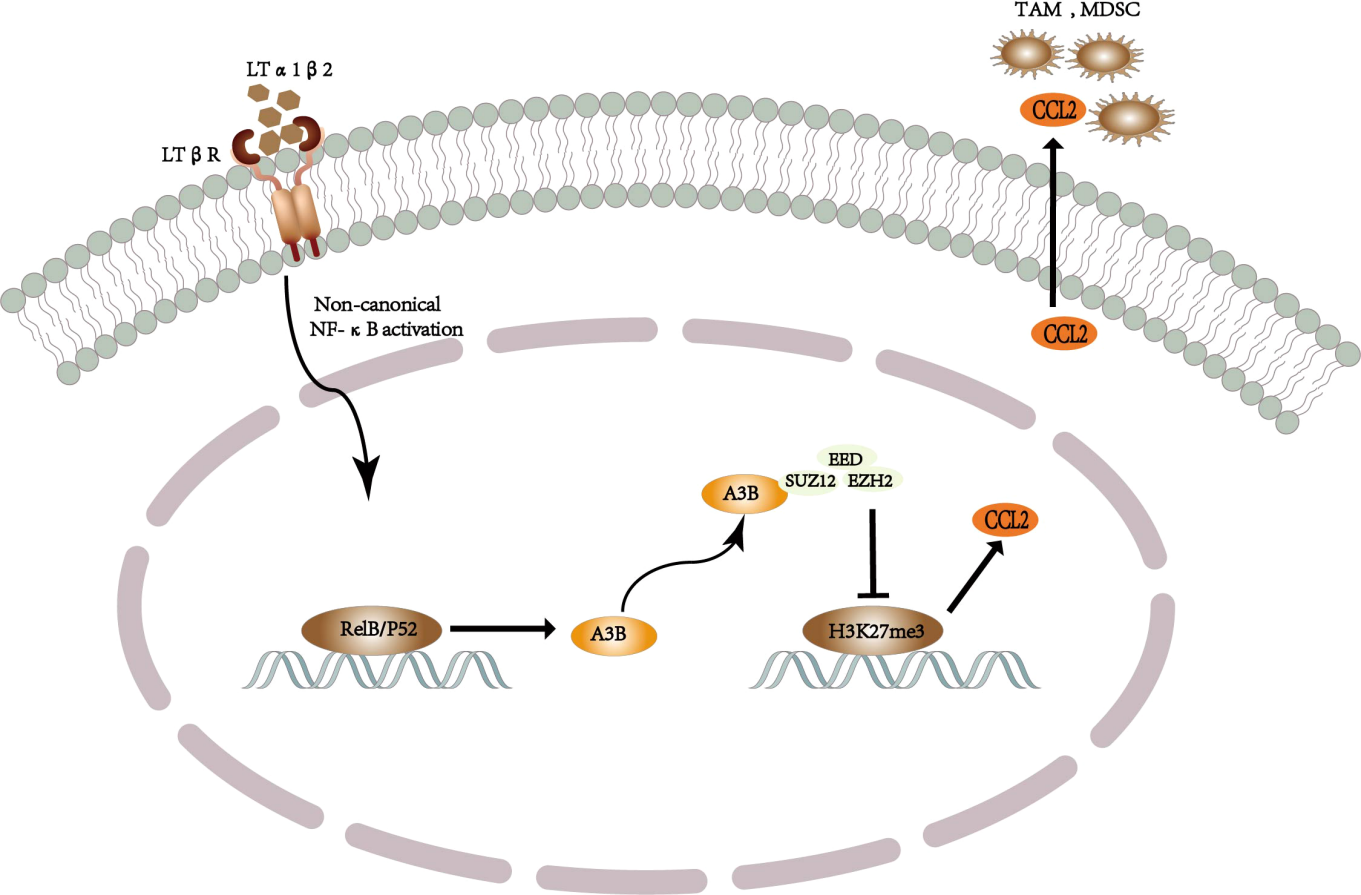
Schematic diagram of the mechanism by which APOBEC3B-driven TAM and MDSC recruit to promote the development of liver cancer.

Duowei et al. explored how APOBEC3B promotes the upregulation of chemokine expression and thus the recruitment of MDSCs and TAMs to promote the occurrence and development of liver cancer; they reviewed previous studies and found that chemokine expression is regulated by genetic and epigenetic mechanisms inherent in cancer, such as DNA methylation and polycomb repressive complex 2 (PRC 2) (82). Furthermore, PRC2 regulates the expression of certain genes by participating in H3K27 methylation (83). In addition, studies have also shown that inhibiting the expression of histone H3 lysine 27 trimethylation (H3K27me3) promotes the upregulation of chemokines such as CCL2, IL-8, and CCL-2, in breast cancer (84), thereby establishing and validating a molecular mechanism for APOBEC3B as an immunomodulatory factor to regulate chemokine expression (81). In this mechanism, APOBEC3B binds to the three core proteins of PRC2, namely, EED, EZH2, and SUZ12, to inhibit PRC2 HMT activity, thereby inhibiting H3K27me3 to upregulate the expression of chemokines, including CCL2, IL-34, and BMP7 (81) (**Figure 3**). Among these chemokines, CCL2 has been established to play a key role in the occurrence and development of liver cancer by aggregating monocytes and macrophages into tumor tissue as well as stimulating tumor cell survival and immune escape (85). In addition, previous studies have shown that EZH2 deficiency promotes cancer formation and increases inflammatory factors to exacerbate the inflammatory response (86, 87). Therefore, the non-enzyme-dependent function that interferes with APOBEC3B inhibits the immunosuppressive microenvironment mediated by a variety of chemokines, thereby inhibiting the occurrence and development of liver cancer.

The high expression of APOBEC3B in liver cancer has been validated, but it remains unclear whether it is upregulated in liver cancer as a lagging upregulation. It has been established that APOBEC3B plays a key role in liver cancer. APOBEC3B overexpression promotes the development of liver cancer *in vivo* and *in vitro*, while APOBEC3B knockout inhibits the development of liver cancer (76). Thus, regulating APOBEC3B is a potential treatment strategy for liver cancer. Moreover, in the APOBEC family, APOBEC1, AID, and APOBEC3B are also closely related to cancer (1), but their related molecular mechanisms still need to be further explored. The present, review of the molecular mechanisms associated with APOBEC3B in liver cancer provides a new possibility for these mechanisms.

## Discussion

In summary, APOBEC3B plays different roles at various stages of liver cancer formation, either by restricting viral replication and transcription through enzyme-dependent functions at the stage of viral infection, or by recruiting MDSCs and TAMs in the tumor microenvironment through non-enzyme-dependent functions in terms of cancer drivers and initiators to stimulate tumor cell survival and immune escape. Although there are still few reports of APOBEC3B in the occurrence and development of liver cancer, it has been established that APOBEC3B plays a key role in liver cancer. Thus the occurrence of liver cancer may be mediated by treatment with APOBEC3B agonist therapy at the HBV stage by restricting viral replication or inhibiting the development of liver cancer by interfering with the non-enzyme-dependent function of APOBEC3B in the early stages of liver cancer. In addition, specific mutation signals and the role of APOBEC3B, as a cytidine deaminase, have been established in the occurrence and development of cervical cancer, breast cancer, lung cancer, and other cancers. Although the mutation signals found in liver cancer do not appear to affect genomic DNA, there may be other roles of the nuclear-localized APOBEC3B cytidine deaminase in hepatocellular carcinoma cells, thus requiring additional exploration.

## Author contributions

XY and JD made substantial contributions to the conception and design of the manuscript. SY, JA, HJ, LZ, LMZ, XC, ZY and BT were involved in revising the manuscript critically for important intellectual content. All authors contributed to the article and approved the submitted version.

## Funding

The collaborative Innovation Center of Chinese Ministry of Education (2020-39), the Science and Technology Plan Project of Guizhou Province (QIAN KE HE JI CHU-ZK (2021) YI BAN 451), the National Natural Science Foundation of China (NSFC, 82160112), the National Natural Science Foundation of China (grant nos.81960507), the Science and Technology Bureau fund of Zunyi city (grant no. ZUN SHI KE HE HZ ZI (2019)93-HAO)

## Conflict of interest

The authors declare that the research was conducted in the absence of any commercial or financial relationships that could be construed as a potential conflict of interest.

## Publisher’s note

All claims expressed in this article are solely those of the authors and do not necessarily represent those of their affiliated organizations, or those of the publisher, the editors and the reviewers. Any product that may be evaluated in this article, or claim that may be made by its manufacturer, is not guaranteed or endorsed by the publisher.
